# *Gigaspora margarita* and Its Endobacterium Modulate Symbiotic Marker Genes in Tomato Roots under Combined Water and Nutrient Stress

**DOI:** 10.3390/plants9070886

**Published:** 2020-07-14

**Authors:** Matteo Chialva, Luisa Lanfranco, Gianluca Guazzotti, Veronica Santoro, Mara Novero, Paola Bonfante

**Affiliations:** 1 Department of Life Sciences and Systems Biology, University of Torino, Viale P.A. Mattioli 25, I-10125 Torino, Italy; matteo.chialva@unito.it (M.C.); gianluca.guazzotti@edu.unito.it (G.G.); mara.novero@unito.it (M.N.); paola.bonfante@unito.it (P.B.); 2 Department of Agricultural, Forest and Food Science, University of Torino, Largo Braccini 2, I-10095 Grugliasco, Italy; veronica.santoro@unito.it

**Keywords:** arbuscular mycorrhizal fungi, drought, endobacteria, multiple stress, nutrients, stress resilience

## Abstract

As members of the plant microbiota, arbuscular mycorrhizal fungi (AMF) may be effective in enhancing plant resilience to drought, one of the major limiting factors threatening crop productivity. AMF host their own microbiota and previous data demonstrated that endobacteria thriving in *Gigaspora margarita* modulate fungal antioxidant responses. Here, we used the *G. margarita*–*Candidatus* Glomeribacter gigasporarum system to test whether the tripartite interaction between tomato, *G. margarita* and its endobacteria may improve plant resilience to combined water/nutrient stress. Tomato plants were inoculated with spores containing endobacteria (B+) or not (B-), and exposed to combined water/nutrient stress. Plants traits, AM colonization and expression of AM marker genes were measured. Results showed that mycorrhizal frequency was low and no growth effect was observed. Under control conditions, B+ inoculated plants were more responsive to the symbiosis, as they showed an up-regulation of three AM marker genes involved in phosphate and lipids metabolism compared with B− inoculated or not-inoculated plants. When combined stress was imposed, the difference between fungal strains was still evident for one marker gene. These results indicate that the fungal endobacteria finely modulate plant metabolism, even in the absence of growth response.

## 1. Introduction

Microbial communities associated with plants—the plant microbiota—include beneficial microorganisms which potentially help their host to cope with biotic and abiotic stresses [1,2]. A role in plant resilience to drought, one of the main current threats for crops, has been attributed to specific components of the root microbiota [2] such as plant growth-promoting bacteria [3,4] and arbuscular mycorrhizal fungi (AMF). The latter are one of the main components of plant mycobiota [5] and may enhance plant resilience to drought, improving nutrients accumulation, water uptake and plant growth [6]. Experimental evidence of such a beneficial role has been obtained in crops including maize [7], soybean [8], wheat [9], date palm [10], apple [11] and tomato [12,13]. AMF are able to efficiently extract water from soil through an extended extra-radical mycelium, to enhance plant stomatal conductance [14] and to induce the plant production of osmolytes. Moreover, they induce the plant production of ROS-scavenging anti-oxidant compounds [15], reducing oxidative stress [16], which is one of the negative effects originated by drought [17]. However, contrasting data emerged from a detailed transcriptomic analysis of two drought-tolerant sorghum cultivars grown under field conditions. Drought stress induced a disruption in the plant AM symbiosis with a corresponding loss of fungal biomass and a significant reduction in symbiosis markers gene expression [18]. These partly contrasting results open many questions on the functional network established between plants and AMF under drought.

A further element of complexity is that AMF host their own bacterial microbiota both at the extra-radical mycelium surfaces and within their cytoplasm, where bacteria live as obligate endobacteria [5]. Among AMF which host endobacteria [19,20], *Gigaspora margarita* with its *Candidatus* Glomeribacter gigasporarum (*Ca*Gg) offers a unique system, since a cured line, without endobacteria, is available [21]. This system offers the unprecedented chance to investigate the role of AMF–endobacteria interactions by comparing the two isogenic lines upon application of diverse “omics” approaches [22,23]. These previous works demonstrated that the obligate endobacterium *Ca*Gg enhances fungal fitness by priming mitochondrial and antioxidant metabolism [22,24]. Superoxide dismutase (Cu/Zn), glutathione peroxidase and thioredoxin reductase resulted as the most abundant proteins in the fungal line hosting the endobacterium [24], mirrored by a highest amount of glutathione and glutathione disulfide [25]. Remarkably, the higher antioxidant capacity of the *Ca*Gg-containing fungal line could also help the host plant to maintain cellular redox homeostasis during symbiosis, lowering the amount of carboxylated proteins, which are markers of oxidative stress [24].

In this context where the molecular mechanisms previously identified in *G. margarita* containing the endobacterium are mainly related to an activation of antioxidant metabolism, we hypothesized that *Ca*Gg could act as an intracellular component of the plant mycobiota and play an active role in plant protection against drought and nutrient stress. We therefore investigated the tripartite interaction between *G. margarita*–*Ca*Gg and tomato plant, using the fungal strain without *Ca*Gg as a control. Tomato (*Solanum lycopersicum*) was used as a model plant being also one of the major horticultural species worldwide. The interaction between tomato and *G. margarita* has been investigated in some previous researches [26,27] which revealed a low mycorrhizal success (around 20–30%). However, considering that (i) different tomato cultivars could differently respond to AM symbiosis, (ii) the amount of colonization is not always considered the major determinant of plant performance [28,29] and (iii) the uniqueness of the *G. margarita*–*Ca*Gg model—we chose tomato as the reference plant. In addition, tomato has extensively been studied to investigate the effect of AM symbiosis upon plant stresses [12,13,30,31,32].

It is known that plants’ responses to multiple abiotic stresses do not fully overlap with plant responses to a singly applied stress [33]. While tomato responses to drought have been largely described [30,31], its response to nutrient stress is well exemplified by the fact that tomato plants grown under low phosphate (Pi) availability showed delayed flowering and fruiting [34], confirming that nutrient limitation has a broad impact on major developmental processes.

Here, we considered a combined water/nutrient stress as this could represent a more realistic condition found in today’s fields and is rarely investigated under laboratory conditions.

## 2. Results

### 2.1. Tomato Plants Inoculated with G. Margarita Showed Low Root Colonization Levels

Tomato plants were inoculated with *G. margarita* spores containing endobacteria (B+) or cured spores without endobacteria (B-), while not-inoculated plants were used as non-mycorrhizal controls (NM). All the plants were grown under normal growth conditions (well-watered, WW) and under combined stress (CS) where a reduction of about 60% of water and nutrients supply was applied.

**Figure 1:**
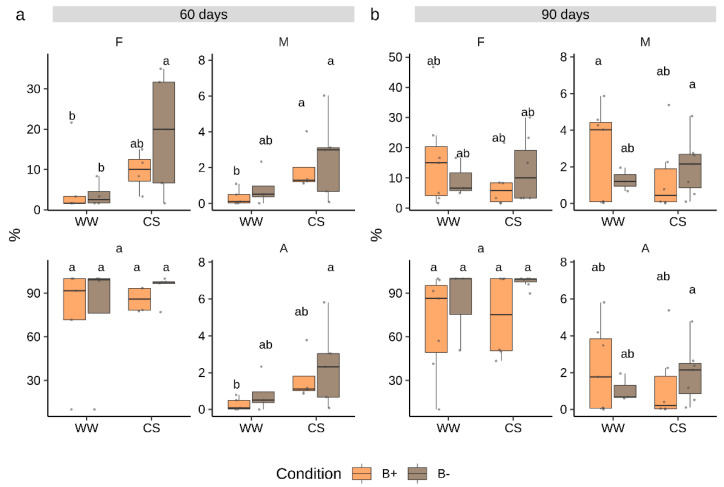
Colonization parameters in *Solanum lycopersicum* cv. M82 inoculated with *Gigaspora margarita* at 60 (**a**) and 90 (**b**) days. Statistically supported differences between mycorrhizal (B- and B+), stress conditions and time points are indicated with different letters (Kruskal–Wallis test, *p* < 0.05). F% = frequency of mycorrhization, M% = intensity of mycorrhization, a% = arbuscules abundance in colonized fragments, A% = arbuscules abundance in whole root apparatus. Boxplots display median value (horizontal line), quartiles (boxes) and 1.5 × interquartile ranges (whiskers).

Since the dynamics of tomato root AM colonization by *G. margarita* were unknown, we sampled plants at 60 and 90 days after inoculation. At each time point, mycorrhizal colonization as well as plant biometric traits were measured. Results showed that *G. margarita* successfully colonized tomato roots mostly producing intracellular hyphae and intercalary arbuscules recalling a Paris-type colonization [26,35]. The colonization only reached relatively low frequency levels at both time points (Figure 1) with a mean frequency (F%) of 15% and 23% at 60 and 90 days, respectively. Similarly, root cortex colonization (M%) showed very low values. By contrast, the abundance of arbuscules in colonized root portions was almost 100% (Figure 1, Figure S1). A few differences between time points, stress treatment and endobacteria presence in any of the measured mycorrhization parameters emerged (ANOVA, *p* > 0.05, Table S1). Notably, B− colonized roots showed a significantly higher frequency of mycorrhization (F%) under combined stress conditions at 60 days, but such a difference was not any more evident at 90 days (Figure 1).

### 2.2. Combined Water and Nutrients Stress Has a Negative Impact on Tomato Growth which is not Alleviated by Mycorrhization

**Figure 2:**
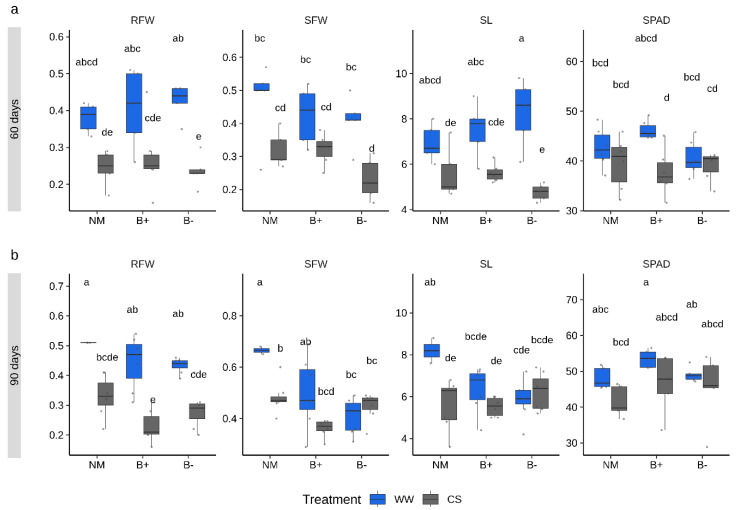
Plant traits measured in tomato inoculated with *G. margarita* containing or not its endobacteria (B+ and B−, respectively) under combined stress (CS, gray) or well-watered (WW, blue) conditions sampled after 60 (**a**) and 90 (**b**) days. Differences between conditions, stress treatment and time points are indicated for each parameter with different letters according to Tukey’s HSD (honestly significant difference) post hoc test after ANOVA (*p* < 0.05). CS, combined stress, WW, normal control conditions. RFW, root fresh weight (g), SFW, shoot fresh weight (g), SL, shoot length (cm), SPAD index. Boxplots display median value (horizontal line), quartiles (boxes) and 1.5 × interquartile ranges (whiskers).

To monitor growth responses under combined stress and mycorrhizal conditions, three biometric parameters (shoot length, SL, shoot fresh weight, SFW, root fresh weight, RFW) and the SPAD (Soil Plant Analysis Development) index, which quantifies the leaf chlorophyll content as a proxy of the plant nutritional status [36], were measured at both sampling time points. ANOVA analysis was performed to detect the effect of each of the four factors within the experimental system (combined stress, mycorrhizal colonization, endobacteria presence, time point and their interactions) on each trait. Results showed that all measured plant traits were mostly modulated by the combined stress treatment (ANOVA, *p* < 0.001; Table 1) and by the time point factor (ANOVA, *p* < 0.05). By contrast, the mycorrhizal colonization factor (considering both *G. margarita* lines) was significant only for SFW (*p* < 0.001), while endobacterial occurrence happened for none of the parameters. However, the interaction effects of combined stress, time point and endobacteria occurrence was significant for SL and SFW. Under CS conditions, both control and mycorrhizal plants accumulated a slightly lower fresh biomass when compared to the well-watered condition, with more marked differences at 90 days (Figure 2). Some responses to CS conditions significantly differed in the B+ and B− lines. In particular, lower values, although not significantly different, were measured in the B− lines at 60 days for the RFW, SFW and SL parameters, while the trend was reversed at 90 days (Figure 2). Irrespective of the time point considered, SPAD index values were similar across conditions, highlighting a comparable nutritional status. This was further confirmed by the analysis of the shoot phosphorus (P) content which showed that the mycorrhizal status did not improve the P content irrespective of the imposed combined stress (Figure S2). The shoot P level, however, displayed a weak increase under CS conditions in all plants.

**Figure 3:**
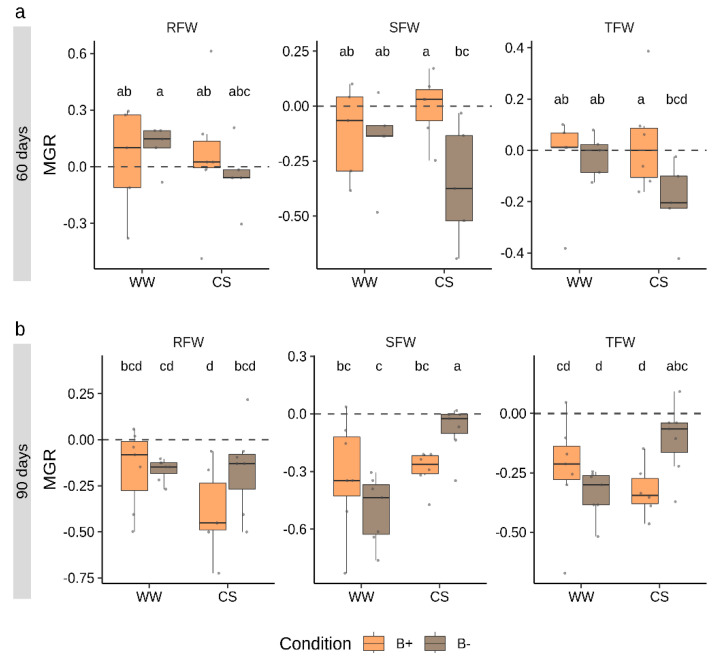
Mycorrhizal growth response (MGR) calculated on biomass parameters in tomato plants colonized by B+ ad B− *G. margarita* lines at 60 days (**a**) and 90 days (**b**) after inoculation under combined stress (CS) and well-watered (WW) conditions. RFW, SFW and TFW refer to MGR values calculated on root, shoot and total fresh weight, respectively. Different letters indicate statistically supported differences, also considering the two time points, according to Kruskal–Wallis test (*p* < 0.05). Boxplots display median value (horizontal line), quartiles (boxes) and 1.5 × interquartile ranges (whiskers).

To track the effects of mycorrhizal colonization in plant fresh biomass accumulation, the mycorrhizal growth response (MGR) parameter was calculated (Figure 3). This index is calculated as the logarithmic ratio between parameters measured in mycorrhizal vs. non-mycorrhizal plants. The analysis was performed for RFW and SFW as well as for total fresh weight (TFW). Data revealed that, while at 60 days, the response to mycorrhization was near to neutrality (MGR = 0), at 90 days, MGR values were negative (MGR < 0) for all the parameters considered. No differences emerged between B+ and B− inoculated plants under WW conditions, while significant differences emerged in MGR calculated on SFW and TFW under CS conditions.

At 60 days, MGR values (SFW and TFW, Figure 3) were significantly higher in B+ than in B− inoculated plants-, while at 90 days, results were reversed with higher responsiveness to mycorrhization in B− colonized plants.

### 2.3. Plant Genes, Marker of a Functional AM Symbiosis, are Modulated by CaGg

To check whether *Ca*Gg endobacterial populations hosted in *G. margarita* had an impact at the molecular level on plant and fungal metabolism, we tested the expression of some genes involved in different aspects of AM symbiosis by using qRT-PCR on materials sampled 90 days after inoculation (Table S2).

**Figure 4:**
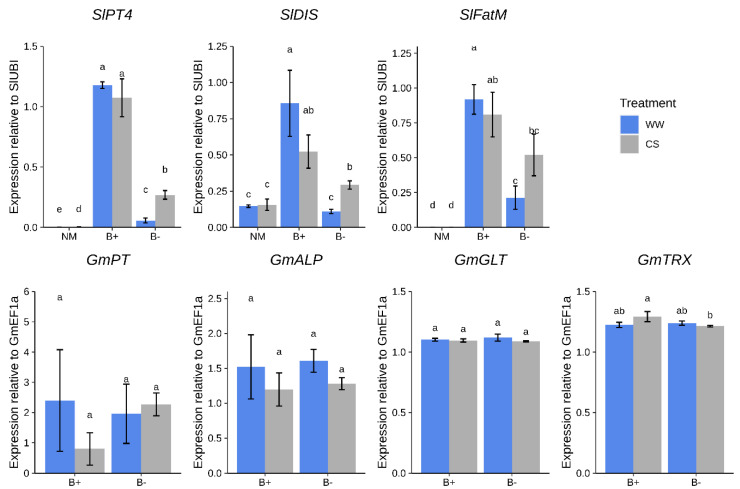
Expression profiles of marker genes in tomato roots colonized by *G. margarita* at 90 days after inoculation. Differences are indicated with different letters according to Kruskal–Wallis test (*p* < 0.05). CS = combined water/nutrient stress, WW = normal conditions. Plant genes: *SlPT4, SlDIS; SlFatM; G. margarita* genes: *GmPT*, *GmALP*, *GmGLT, GmTRX.*

First, we measured the expression of plant genes involved in nutrients exchange such as those involved in AM-dependent phosphate transport (*Sl*PT4, [37]) and lipids biosynthesis (*Sl*FatM and *Sl*DIS, [38,39]). While the first indirectly indicates the occurrence of phosphate transfer from the AMF towards the plant, the other two can be considered markers of the lipids transfer from the plant towards the AMF, which are indeed fatty acid auxotrophs [40,41]. Notably, under well-watered conditions (WW), these transcripts were more abundant in B+ vs. B− colonized roots (*p* < 0.05, Figure 4).

Under CS conditions, while differences in *SlPT4* remained, the expression of *SlDIS* and *SlFatM* significantly increased in B− roots, reaching levels similar to those found in B+ mycorrhizal roots.

As a second step, we checked the abundance of *G. margarita* fungal transcripts involved both in symbiosis-dependent phosphate metabolism and endobacterium-mediated ROS metabolism [22]. No relevant difference between the two fungal strains was detected looking at the expression levels of the fungal phosphate transporter *GmPT* and alkaline phosphatase *GmALP* genes (Figure 4). Both genes were found to be expressed in arbusculated cells [41]. We also tested two fungal markers of ROS detoxification, a glutathione peroxidase (*GmGLT*) and a thioredoxin reductase (*GmTRX*), since they were abundantly expressed and up-regulated in the pre-symbiotic phase of the B+ compared with the B− fungal line [22,24]. Notably, while *GmGLT* did not show differential regulation under any condition, *GmTRX* was slightly up-regulated under CS conditions in tomato B+ roots compared with B− inoculated plants (Figure 4). Taken in their whole, the experiments reveal that the plant and not only the fungus, perceives the presence of the endobacterium hosted inside the fungal cytoplasm.

## 3. Discussion

Several studies showed the potential of AM symbiosis to alleviate nutrients and drought stress in tomato plants [12,13,31,32,34]. These evidences highlight that application of AMF under environmental stress conditions in tomato deserves further experimental validation by testing additional host and AMF genotypes.

### 3.1. The Combination G. Margarita/Tomato cv “M82” Does not Exploit the Benefits of the Symbiosis

In this work, we used *G. margarita–Ca*Gg—at the moment the unique available system which allows to manipulate the endobacterial presence in AMF—to disentangle the role of endobacteria in modulating plant responses to a combined water/nutrient stress. We selected to grow the plants in small alveolar trays as we could mimic the large-scale growing conditions used to produce tomato seedlings in industrial nurseries and we could easily apply the combined stress.

Our findings first showed that under controlled experimental conditions, *G. margarita* led to a low and patchy mycorrhizal colonization without evidence of a growth effect. The observed colonization rate (around 20%) was similar to that reported by Tahat et al. [27] on a different tomato genotype colonized by *G. margarita*, and much less than the mycorrhizal frequency (typically higher than 60–80%) obtained by inoculating the AMF fungus *F. mosseae* [12,42], even in the same alveolar tray system used in this study (data not shown). Probably due to their huge genome, which requires energetic investments for multiplication [23], Gigasporaceae are not equally successful in colonizing roots of all plant species, and in particular those which, as tomato, need important nutrient support [34]. These considerations are also mirrored by ecological data: indeed, a global assessment of AMF diversity [43] showed that Gigasporaceae have a limited ecological distribution when compared with other AMF families such as Glomeraceae.

### 3.2. The Endobacterium Living in G. Margarita Modulates Molecular Responses of Tomato

Our data showed that the presence/absence of the endobacterium had no impact on mycorrhization, confirming previous results [21].

The measurements of growth-related parameters and SPAD revealed that the positive effect of *G. margarita* and its bacterial endosymbiont on plant responses was limited, possibly as a consequence of the low root colonization, and that major differences were found at 60 days after inoculation. The endobacterium seems to have a slight time-dependent effect on plant growth parameters. This probably mirrors the colonization dynamics of the B− fungal strain which is characterized by a slower growth rate compared with B+ [21]. Overall, the complex *G. margarita*–*Ca*Gg did not enhance tomato growth under combined stress, which caused the greatest negative impact at both time points. Accordingly, when analyzing SPAD index values and P shoot content, we found no differences between mycorrhizal and non-mycorrhizal plants under both control and CS conditions. Similarly, Bulgarelli et al. [44] did not find any increase in P shoot level in soybean upon mycorrhization and, more in general, they found no correlation between P accumulation and growth responses, probably due to the fact that soybean has a high P demand, such as tomato. Moreover, under CS treatment, a slight increase in the P shoot content was observed, and this was probably caused by the specific experimental conditions which enhance the tomato attitude to accumulate P in the epigeous organs [45].

Notwithstanding the low colonization rate and the absence of a growth effect of *G. margarita* on tomato, the molecular mechanisms associated with arbuscule functioning were activated. Three plant marker genes, at the basis of the reciprocal benefits in AM symbiosis, were up-regulated in mycorrhizal plants compared with non-mycorrhizal plants, even under combined CS stress, as also described by Volpe et al. [30]. An opposite behavior, i.e., mycorrhizal marker genes strongly down-regulated under drought conditions, was described for sorghum plants [18]: this discrepancy could be explained by differences in the amplitude of the water-stress imposed (severe vs. moderate), in the growth conditions (open-field vs. controlled growth chamber) and in the plant species used (monocot vs. dicot). Another explanation may lay in that the native sorghum-associated AMF are probably not well-adapted to drought conditions.

Interestingly, especially in the WW conditions, plants colonized by the fungus with its endobacterium (B+) showed a stronger up-regulation of plant symbiotic genes compared with B− inoculated roots. Among the symbiotic marker genes, *PT4* revealed a significant up-regulation, notwithstanding the unchanged P amount detected in the shoot.

Probably as a consequence of the limited colonization success, *G. margarita* did not provide advantages in terms of vegetative biomass, irrespective of the growth conditions and of the activation of the phosphate transporters in both the fungus and the plant. The results also indicate that the presence of endobacteria living inside the fungus finely modulate plant metabolism through the regulation of AM symbiotic genes.

Taken as a whole, tomato growing in the alveolar trays demonstrated to perceive the difference between *G. margarita* with and without its endobacterium, and to activate the signaling and the accommodation process, but these cellular and molecular events were not sufficiently powerful to elicit a growth effect and stress tolerance. Even if the leading hypothesis (whether an endobacterium increases plant resistance to environmental stress) was not confirmed, the investigation provides novel information on the molecular mechanisms underlying AM establishment: even in the presence of a reduced fungal biomass, the plant distinguishes between the two fungal lines and, accordingly, modulates the expression of two genes (the mycorrhiza-inducible phosphate transporter *SlPT4* and *SlFatM*) which are two iconic markers of AM functionality.

## 4. Materials and Methods

### 4.1. Plant Materials, Experimental Set-Up and Sampling

To mimic the large-scale growing conditions which are used to produce tomato seedlings in industrial nurseries, the experiment was set-up in a miniaturized system constituted by small alveolar trays (60 wells, 5 × 5 × 14 cm and 100 mL in volume each) filled with oven-sterilized (180 °C, 3 h) quartz sand. Tomato (*Solanum lycopersicum* cv “M82” LA3475) seeds were surface-sterilized with a 3 min wash in 70% ethanol with the addition of 3 drops of Tween 20 (3 min) followed by a wash in 2.5% sodium hypochlorite in sterile dH_2_O (13 min) and three rinses in sterile dH2O (10 min each). Seeds were plated in 0.6% plant agar medium (Duchefa, Haarlem, The Netherlands) and germinated in the dark at 23 °C for five days and then moved to day/night conditions for at least four days (16 h/8 h light/dark photo-period). The experimental set-up consisted of 2 mycorrhization conditions (B+ and B−) plus an uninoculated control, and a combined water/nutrient treatment (CS) with its respective untreated control (WW). Two sets of 6 plants each were prepared: the first to be sampled at 60 days after inoculation and the second at 90 days after inoculation.

At the end of the preliminary growth phases, for each experimental condition, 12 seedlings, for a total of 72 individuals, were then transferred to alveolar trays and parts of the seedlings were inoculated using 100 spores/plant of *Gigaspora margarita* Becker and Hall (isolate BEG 34, deposited at the European Bank of Glomeromycota), containing *Ca*Gg endobacteria (B+) or spores of a cured line without endobacteria (B-; [21]). *G. margarita* spores were propagated in *Trifolium repens* trap cultures, isolated using the wet sieving technique [46] and manually collected. Spores were then surface-sterilized in 3% *w/v* chloramine-T and 0.03% *w/v* streptomycin sulfate and subsequently washed with sterile water three times for 10 min each. Spores were then deposited around roots while potting seedlings in sterile quartz sand. Control plants were grown on the same substrate but no *G. margarita* spores were added. The experimental set-up is summarized in Figure S4.

Plants were grown in a controlled growth chamber (14 h light at 24 °C/10 h dark at 20 °C) and watered twice a week with a modified Long Ashton solution [47] containing 3.2 μM Na_2_HPO_4_, and 1 mM NaNO_3_. A low P content was used to ensure good levels of root colonization since a higher concentration could impair AM symbiosis establishment. After the first 30 days in which AM symbiosis could establish without interferences [9,48], combined stress was gradually induced by lowering the substrate water capacity (SWC%) as described in Lehnert et al. [9]. Untreated plants (WW conditions) were watered with Long Ashton solution as described above, allowing to compare them with plants in which both nutrients and water were lowered to induce a combined water/nutrient stress (CS conditions). The weight of the alveolar trays was monitored each two days and maintained at >90% in well-watered control plants and at 35% in stressed plants. These values allowed to maintain the stem water potential between −0.6 and −0.8 MPa in the combined stress treatment (moderate water stress) and higher than −0.5 MPa in controls as established in a preliminary pilot experiment (data not shown). Similarly, these conditions allowed to reduce 60% of nutrients supply to CS plants inducing combined stress.

To confirm that a moderate water stress was achieved, the stem water potential was monitored at the end of the experiment on three randomly sampled plants for each tray and the SWC% levels confirmed (Figure S3).

Shoot water potential was indirectly measured from leaves according to Freitas et al. [49] Briefly, leaves were enclosed in plastic bags with wet filter paper and a reflective envelope to suppress transpiration allowing the leaf water potential to equilibrate with the stem. After 20 min, leaves were excised from the stem and shoot water potential was measured using a Schölander pressure chamber.

AM colonization dynamics were followed sampling 6 plants per condition after 60 days and the remaining 6 at 90 days after the start of the experiment. During each harvesting, plants were sampled and roots washed thoroughly under tap water to remove sand particles. Root/shoot fresh weight (RFW and SFW) and shoot length (SL) were measured. During the experiment, SPAD index was measured after the first month and at both sampling points using an SPAD-502 Chlorophyll Meter (Konica-Minolta Inc., Chiyoda, Tokyo, Japan). For each plant, the values recorded from three different leaflets of the first fully expanded leaf from apical meristem were considered. The mycorrhizal growth response parameter (MGR) on RFW, SFW and total fresh weight (TFW) was calculated, according to Hoeksema et al. [50] as reported in Equation (1).

(1) MGR = log_e_(TFW_MYC/_TFW_NM_)

Mycorrhizal colonization was estimated on a representative portion of root materials at each sampling time point. Roots were stained in 0.1% cotton blue Sigma-Aldrich (St. Louis, MO, USA), bleached in lactic acid (two washes, 1 h each) and 1 cm-length fragments assembled on glass slides. Roots segments (60 cm per plant) were then observed and AM colonization scored according to Trouvelot et al. [51]. To exclude cross-contamination, mycorrhizal colonization was also checked on non-mycorrhizal plants (NM).

Total phosphorus content was determined colorimetrically using the malachite green method [52], after sulfuric-perchloric digestion [53] on ground, oven-dried (48 h at 40 °C) shoot material.

### 4.2. RNA Isolation and Processing

RNA was isolated from 3 different plants for each condition, starting from freeze-dried root tissue using NucleoSpin^®^ RNA Plant and Fungi kit (Macherey-Nagel, Düren, Germany) and quantified by using a NanoDrop spectrophotometer (NanoDrop Technologies, Inc., Wilmington, DE, USA). Samples were then diluted to 200 ng/ul and subjected to a DNAse treatment using the Turbo DNA-freeTM kit (Ambion, Austin, TX, USA) according to the manufacturer’s instructions. The absence of DNA contamination was assessed in PCR assays using primers for the tomato ubiquitin gene (Solyc01g056940; [54]. cDNA was synthesized from about 400 ng of DNA-free total RNA using the SuperscriptTM II Reverse Transcriptase Kit (Invitrogen, Waltham, MA, USA) according to the manufacturer’s instructions. cDNAs were 1:2 diluted for quantitative relative expression analysis (RT-qPCR).

### 4.3. Quantitative Real-Time PCR (RT-qPCR)

Reactions were carried out in two technical replicates using a Rotor-Gene Q apparatus (QIAGEN, Hilden, Germany) in a final volume of 15 µL containing 2.25 µL of water, 7.5 µL of Rotor-Gene SYBR^®^ Green PCR Mastermix (QIAGEN, Hilden, Germany), 2.25 µL of 3 µM forward and reverse primers and 0.75 µL of cDNA sample. The PCR cycling program consisted of a holding stage of 10 min at 95 °C and 40 cycles of 15 s at 95 °C and 1 min at 60 °C. A melting curve (55–95 °C) with a heating rate of 0.5 °C for 10 s and a continuous fluorescence measurement was recorded at the end of each run. Oligonucleotides were designed using tomato CDSs as a reference (SL2.5 version from Solgenomics database, http://solgenomics.net) using the primer3 v4.1.0 web software (http://bioinfo.ut.ee/primer3/) and purchased from Sigma-Aldrich (St. Louis, MO, USA) (Table S2). Take-off (C_q_) and amplification efficiency values were calculated using the “comparative quantitation” mode in the Rotor-Gene Q software. Normalized relative gene expression was calculated using ubiquitin (*UBI*) and elongation factor 1α (*EF1α*) as a reference gene for tomato and *G. margarita*, respectively [54,55]. Normalized relative quantities (NRQ) of gene expression were calculated based on gene specific amplification efficiencies using the sample with the lower C_q_ value as the calibrator, as described in Pfaffl [56]. Statistical analysis was performed on log_2_-normalized NRQ values using a pairwise Kruskal–Wallis test as described below.

### 4.4. Data Analysis

Statistical tests were performed in the R statistical programming environment [57] using RStudio GUI v1.2.5019 [58]. Data were log_2_-transformed and normality and homoscedasticity were tested using Shapiro–Wilk [59] and Levene’s tests [60] in the “stats” v3.6.3 and “car” v3.0-7 R packages [61], respectively (*p* < 0.05). According to data distributions, ANOVA or aligned rank transformed (ART) was performed on raw values for parametric and non-parametric data, respectively [62], using the R packages “stats” and “ARTool” v10.0.7, respectively (*p* < 0.05). The endobacteria presence/absence factor was modeled as nested in the mycorrhization factor. Pairwise comparisons between treatments were performed using the appropriate post hoc tests. Tukey’s test [63] and pairwise Kruskal–Wallis [64] tests from the package “agricolae” v1.3.2 [65] were adopted at *p* < 0.05 for parametric and non-parametric data, respectively. Graphical elaborations were performed using the “ggplot2” v3.3.0 package [66].

## Figures and Tables

**Table 1:** Four-way ANOVA table on plant growth traits data (model formula: parameter ~ treatment × (AM colonization/endobacteria) × time point). SL, shoot length, SFW, shoot fresh weight, RFW, root fresh weight, SPAD index. Significant factors’ effects and their interactions (*p* < 0.05) are shown in bold. CS, combined water/nutrient stress.

		SL		SFW		RFW		SPAD	
Factor	*df*	F	*p*	F	*p*	F	*p*	F	*p*
CS	1	37.058	**0.000**	25.013	**0.000**	97.428	**0.000**	18.124	**0.000**
AM colonization	1	0.972	0.328	15.854	**0.000**	1.2875	0.261	1.586	0.213
Time point	1	1.299	0.257	31.937	**0.000**	6.505	**0.014**	28.480	**0.000**
AM colonization × endobacteria	1	0.043	0.836	1.427	0.237	0.169	0.683	2.871	0.096
CS × AM colonization	1	0.881	0.352	2.630	0.110	0.550	0.461	0.054	0.817
CS × time point	1	10.290	**0.002**	2.636	0.100	0.299	0.587	0.343	0.561
AM colonization × time point	1	3.033	0.087	4.089	**0.048**	6.502	**0.014**	2.503	0.119
CS × AM colonization × endobacteria		0.000	0.993	2.25	0.139	0.136	0.714	3.108	0.084
AM colonization × endobacteria × time point	1	0.117	0.734	1.267	0.265	0.742	0.393	0.063	0.802
CS × AM colonization × time point	1	12.593	**0.001**	2.069	0.156	0.116	0.735	0.359	0.552
CS × AM colonization × endobacteria x time point	1	6.299	**0.015**	7.799	**0.007**	2.645	0.110	0.423	0.518

## Supplementary Materials

The following are available online at https://www.mdpi.com/2223-7747/9/7/886/s1, Figure S1: Micrographs showing cotton-blue staining of AM colonization in tomato roots colonized by G. margarita, Figure S2: Shoot phosphorus content of tomato plants inoculated with G. margarita containing or not its endobacteria and the control (B+, B−, NM) under combined stress (CS, gray) or well-watered (WW, blue) after 90 days, Figure S3: Stem water potential in control (well-watered) and combined-stress (CS) plants collected at 60 and 90 days after inoculation, Figure S4. Experimental set-up scheme. Table S1: Non-parametric three-way ANOVA table on aligned rank transformed (ART) data of mycorrhizal colonization parameters, Table S2: Primer sequences used in RT-qPCR experiments.
