# Supplementary material for: Gigaspora margarita and Its Endobacterium Modulate Symbiotic Marker Genes in Tomato Roots under Combined Water and Nutrient Stress
Source: Plants (Basel). 2020 Jul 14;9(7):886. doi: 10.3390/plants9070886 (PMC7412303; doi:10.3390/plants9070886)
Supplement: Supplementary file 1 [file plants-09-00886-s001.pdf]

Gigaspora Margarita and Its Endobacterium Modulate Symbiotic Marker Genes in Tomato Roots under Combined Water and Nutrient Stress

Matteo Chialva[1], Luisa Lanfranco[1*], Gianluca Guazzotti[1], Mara Novero[1], Paola Bonfante[1]

[1]*Department of Life Sciences and Systems Biology, University of Torino, Viale P.A. Mattioli 25, I-10125 Torino, Italy*

**Corresponding author:* luisa.lanfranco@unito.it, Department of Life Sciences and Systems Biology, University of Torino, Viale P.A. Mattioli 25, I-10125 Torino, Italy, +39 011 670 5969. ORCID: 0000-0002-3961-2552

Supplementary Materials

Supplementary Figures

Figure S1 Micrographs showing cotton-blue staining of AM colonization in tomato roots colonized by *G. margarita*.

Figure S2 Shoot phosphorus content of tomato plants inoculated with *G. margarita* containing or not its endobacteria and the control (B+, B-, NM) under combined stress (CS, gray) or well-watered (WW, blue) after 90 days.

Figure S3 Stem water potential in control (well-watered) and combined-stress (CS) plants collected at 60 and 90 days after inoculation.

Figure S4 Experimental set-up scheme.

Supplementary Tables

Table S1. Non-parametric three-way ANOVA table on Aligned Rank Transformed (ART) data of mycorrhizal colonization parameters.

Table S2. Primer sequences used in RT-qPCR experiments.

Suppelementary Figures

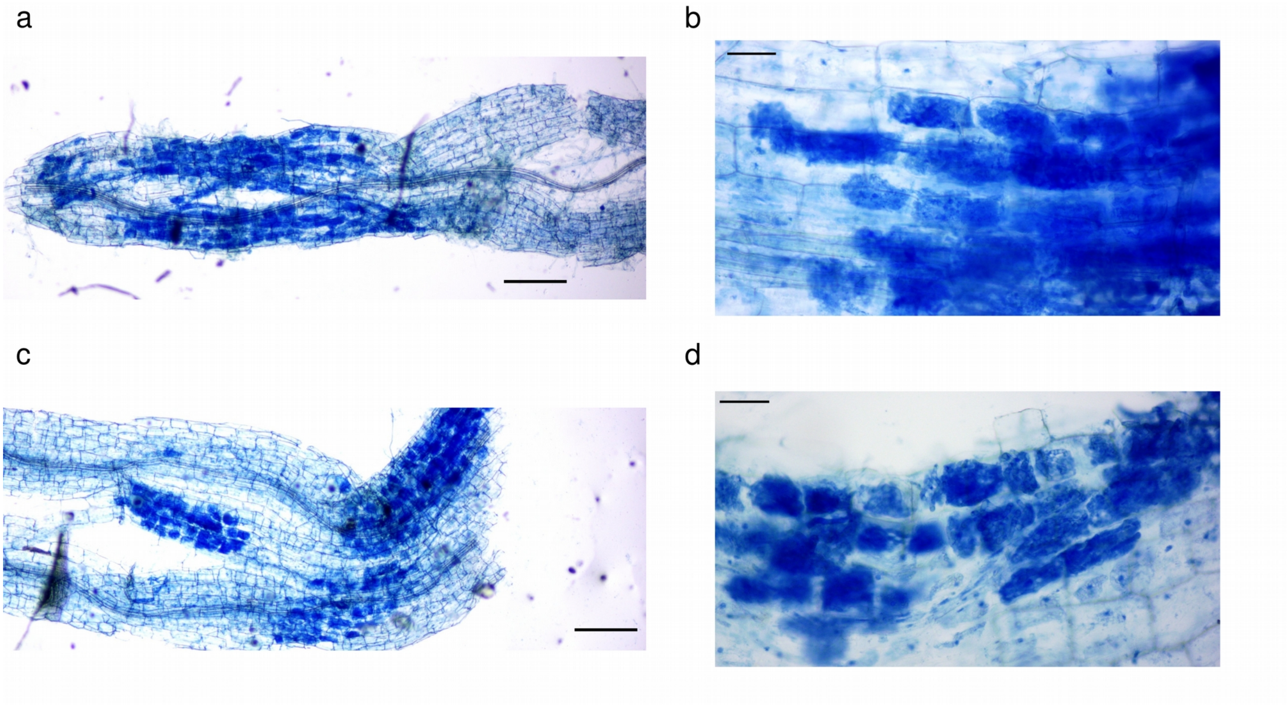

Figure S1 Micrographs showing cotton-blue staining of AM colonization in tomato roots colonized by *G. margarita*. Details of roots colonized by *G. margarita* B- and B+ line are showed in (a-b) and (c-d) respectively. First column pictures are at 10× magnification (scale bars = 100 µm) while in second column pictures are at 20× magnification (scale bars = 50 µm).

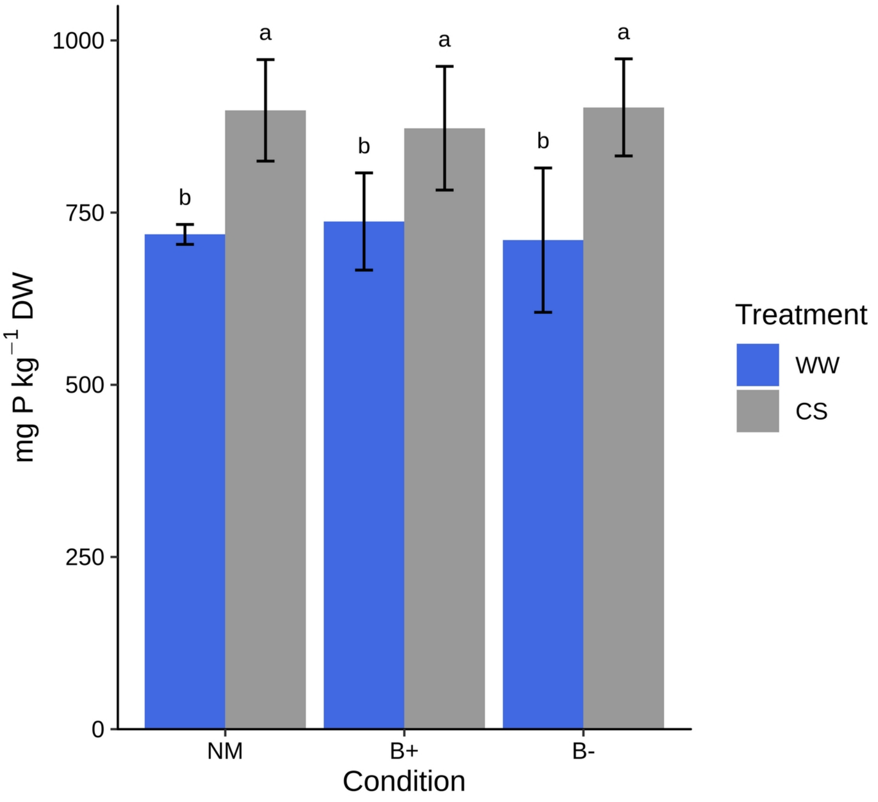

Figure S2 Shoot phosphorus content of tomato plants inoculated with *G. margarita* containing or not its endobacteria and the control (B+, B-, NM) under combined stress (CS, gray) or well-watered (WW, blue) after 90 days. Differences across conditions and treamtents are indicated with different letters according to Tukey's HSD (honestly significant difference) post hoc test after ANOVA (P<0.05).

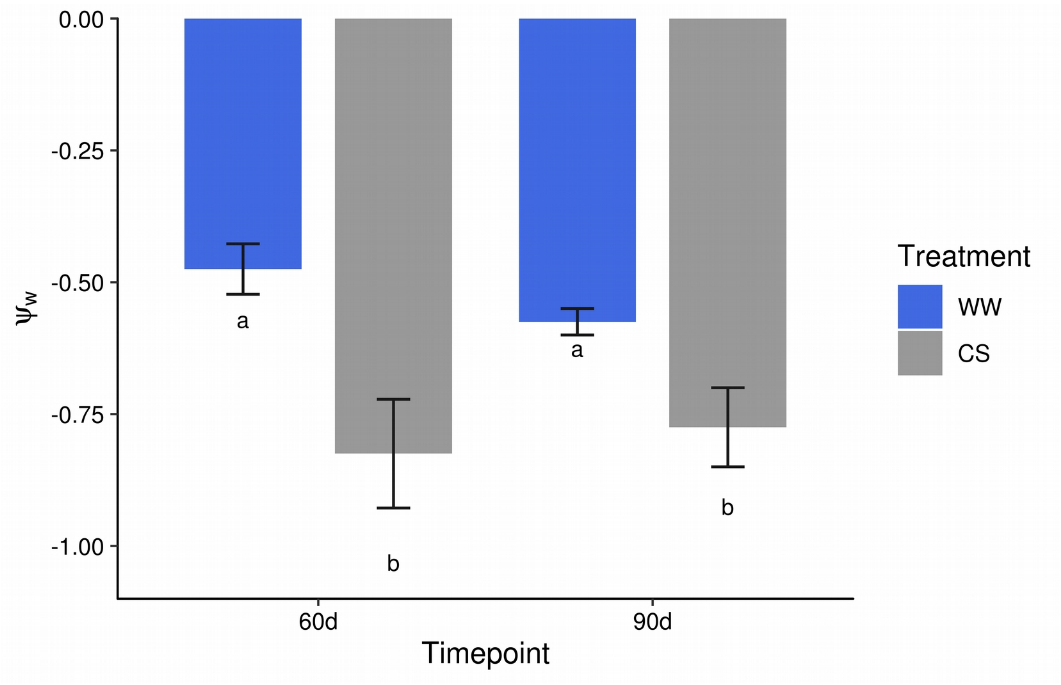

Figure S3 Stem water potential in control (well-watered) and combined-stress (CS) plants collected at 60 and 90 days after inoculation. Different letters indicate statistically supported differences according to the non-parametric Kruskal-Wallis test (P<0.05).

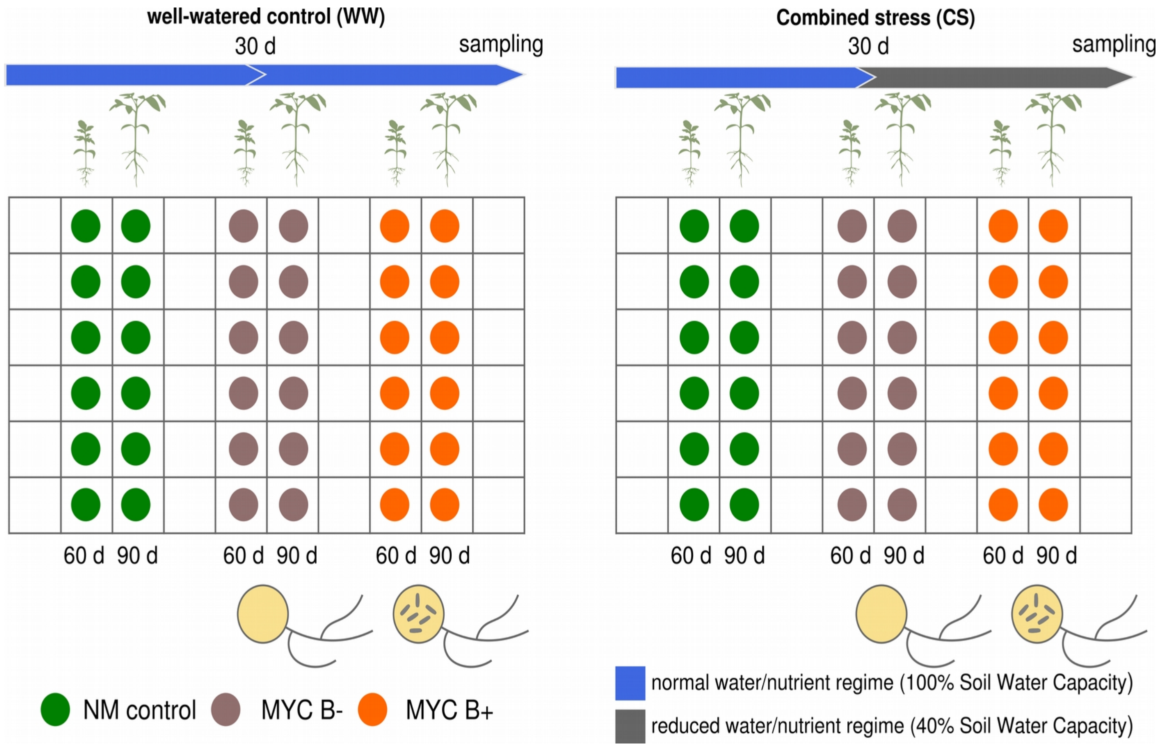

Figure S4. Experimental set-up scheme. Plants were inoculated with *G. margarita* spores (MYC B- and MYC B+) or not-inoculated (NM) into alveolar trays. Combined water-nutrient stress was induced by reducing up to 35% the substrate water capacity (SWC%) of each well after the first month by estimating the mean SWC% of the whole tray by weighting the system. For each condition 12 plants were potted: 6 were sampled at 60 days and the remaining 6 after 90 days.

Supplementary Tables

Table S1. Non-parametric three-way ANOVA table on Aligned Rank Transformed (ART) data of mycorrhizal colonization parameters (model formula: parameter ~ CS × endobacteria × timepoint). None of the factors and their interactions had significant effects on AM colonization parameters (*P*<0.05). CS, combined water/nutrients stress. F%=frequency of mycorrhization, M%=intensity of mycorrhization, a%=arbuscules abundance in colonized fragments, A %=arbuscules abundance in whole root apparatus.

		**F%**		**M%**		**a%**		**A%**	
**Factor**	*df*	*F*	*P*	*F*	*P*	*F*	*P*	*F*	*P*
CS	1	0.746	0.394	0.183	0.672	0.017	0.897	0.758	0.390
endobacteria	1	0.622	0.436	0.139	0.711	4.039	0.053	0.741	0.396
timepoint	1	0.111	0.741	0.756	0.391	0.251	0.620	0.320	0.575
CS × endobacteria	1	2.345	0.135	0.921	0.344	0.161	0.691	0.563	0.458
CS × timepoint	1	3.428	0.073	3.352	0.076	0.115	0.737	2.380	0.132
endobacteria × timepoint	1	0.521	0.475	0.378	0.543	0.391	0.536	0.047	0.830
CS × endobacteria × timepoint	1	0.037	0.849	0.916	0.346	0.001	0.979	0.577	0.453

Table S2. Primer sequences used in RT-qPCR experiments.

Organism	Gene	Annotation	Transcript ID[1]	Forward primer (5’ -3’)	Revers primer (5’ -3’)	Reference
S. lycopersicum	UBI	Ubiquitin	Solyc01g056940.2	ACCAAGCCAAAGAAGATCAAGC	GTGAGCCCACACTTACCACAGT	Fiorilli et al., (2009)
	PT4	Mycorrhiza-inducible inorganic phosphate transporter 4 (*LePT4*)	Solyc06g051850.1	CCGAGACAAAAGGGAGATCAC	CCAGAGACAGGTTTGCTAGTC	Chialva et al., (2016)
	FatM	Acyl-[acyl-carrier-protein] hydrolase	Solyc05g008570.1	AGCCACAGGCCTTGTATTTG	TCCTCTTTGATGGCTTGCTTAC	This study
	DIS	3-oxoacyl-(Acyl carrier protein) synthase II	Solyc08g082620.2	AAATGAACGGGACAAAGTCG	GTTGGATGAAGCCATCCTGT	This study
G. margarita	EF1a	Elongation factor 1-alpha	AJ566401	TGAACCTCCAACCAGACCAACTG	CGGTTTCAACACGACCTACAGGGAC	Salvioli et al., (2008)
	ALP	alkaline phosphatase	AB114299.1	ACCGAATATCTTAACTTGGATCCTG	AAGTTCGGCGAGGTATATGACC	Xie et al., (2016)
	PT	phosphate transporter	KC887075	TCATGGATTAAGTGCAGCCTC	AGAAAACATAAAGATAGCGAAT	Xie et al., (2016)
	GLT	glutathione peroxidase	comp33201_c3	ACGAAATGGCCTGCACATGA	ACTTTCGGGCGTTTGACCAG	Salvioli et al., (2016)
	TRX	thioredoxin reductase	comp37118_c0	GCTGCAGGCGATGTGAAAGA	GCCAACGCTCGCATTCCAAT	Salvioli et al., (2016)

[1] *S. lycopersicum* transcript IDs (SL2.5, ITAG2.4 genome/annotation version) are provided while for *G. margarita* CDSs NCBI accession codes or, where not available, scaffold names of the available transcriptome assembly (Salvioli et al. 2016) are reported.

References

Chialva M, Zouari I, Salvioli A, et al (2016) *Gr* and *hp-1* tomato mutants unveil unprecedented interactions between arbuscular mycorrhizal symbiosis and fruit ripening. *Planta* 244:155–165. https://doi.org/10.1007/s00425-016-2491-9

Fiorilli V, Catoni M, Miozzi L, et al (2009) Global and cell-type gene expression profiles in tomato plants colonized by an arbuscular mycorrhizal fungus. *New Phytologist* 184:975–987. https://doi.org/10.1111/j.1469-8137.2009.03031.x

Salvioli A, Ghignone S, Novero M, et al (2016) Symbiosis with an endobacterium increases the fitness of a mycorrhizal fungus, raising its bioenergetic potential. *ISME J* 10:130–144. https://doi.org/10.1038/ismej.2015.91

Salvioli A, Lumini E, Anca I-A, et al (2008) Simultaneous detection and quantification of the unculturable microbe *Candidatus* Glomeribacter gigasporarum inside its fungal host *Gigaspora margarita* - Salvioli. *New Phytologist* 180:248–257. https://doi.org/10.1111/j.1469-8137.2008.02541.x

Xie X, Lin H, Peng X, et al (2016) Arbuscular Mycorrhizal Symbiosis Requires a Phosphate Transceptor in the *Gigaspora margarita* Fungal Symbiont. *Molecular Plant* 9:1583–1608. https://doi.org/10.1016/j.molp.2016.08.011
